# Using a historic drought and high‐heat event to validate thermal exposure predictions for ground‐dwelling birds

**DOI:** 10.1002/ece3.3185

**Published:** 2017-07-08

**Authors:** James M. Carroll, Craig A. Davis, R. Dwayne Elmore, Samuel D. Fuhlendorf

**Affiliations:** ^1^ Department of Natural Resource Ecology and Management Oklahoma State University Stillwater OK USA

**Keywords:** climate change, *Colinus virginianus*, microclimate, northern bobwhite, temperature, thermal environment

## Abstract

Deviations from typical environmental conditions can provide insight into how organisms may respond to future weather extremes predicted by climate modeling. During an episodic and multimonth heat wave event (i.e., ambient temperature up to 43.4°C), we studied the thermal ecology of a ground‐dwelling bird species in Western Oklahoma, USA. Specifically, we measured black bulb temperature (*T*_bb_) and vegetation parameters at northern bobwhite (*Colinus virginianus*; hereafter bobwhite) adult and brood locations as well as at stratified random points in the study area. On the hottest days (i.e., ≥39°C), adults and broods obtained thermal refuge using tall woody cover that remained on average up to 16.51°C cooler than random sites on the landscape which reached >57°C. We also found that refuge sites used by bobwhites moderated thermal conditions by more than twofold compared to stratified random sites on the landscape but that *T*_bb_ commonly exceeded thermal stress thresholds for bobwhites (39°C) for several hours of the day within thermal refuges. The serendipitous high heat conditions captured in our study represent extreme heat for our study region as well as thermal stress for our study species, and subsequently allowed us to assess ground‐dwelling bird responses to temperatures that are predicted to become more common in the future. Our findings confirm the critical importance of tall woody cover for moderating temperatures and functioning as important islands of thermal refuge for ground‐dwelling birds, especially during extreme heat. However, the potential for extreme heat loads within thermal refuges that we observed (albeit much less extreme than the landscape) indicates that the functionality of tall woody cover to mitigate heat extremes may be increasingly limited in the future, thereby reinforcing predictions that climate change represents a clear and present danger for these species.

## 1 INTRODUCTION

Although comparatively rare, extreme climatic events can have substantial impacts on populations (Easterling et al., 2000; Holmgren et al., 2006; Parmesan, Root, & Willig, 2000). For example, the frequency, severity, and extent of high heat events can dictate species distributions (Jiguet et al., 2006; Parmesan et al., 2000), constrain behavior (Austin, 1976; Cunningham, Martin, Hojem, & Hockey, 2013; Ricklefs & Hainsworth, 1968; Zimmerman et al., 1994), and inhibit physiological performance of organisms (Dawson, 1982; McKechnie, Hockey, & Wolf, 2012; du Plessis, Martin, Hockey, Cunningham, & Ridley, 2012). Even short‐term heat waves have led to catastrophic population‐level mortality events in endothermic bird and bat species in arid regions of Australia and the United States (Finlayson, 1932; Miller, 1963; Towie, 2009; Welbergen, Klose, Markus, & Eby, 2008). Increased heat loads have also been linked to population‐level extinctions in ectothermic lizards in Mexico (Sinervo et al., 2010). In the future, thermal conditions currently considered as extreme events are predicted to become more frequent and extensive in many regions due to climate change (IPCC 2013). Accordingly, extreme heat events can present researchers with an opportunity to evaluate the effects of climate projections on organisms (Boyles, Seebacher, Smit, & McKechnie, 2011; McKechnie et al., 2012); however, doing so typically necessitates either long‐term studies or those that serendipitously capture climatic events (e.g., episodic high heat events).

While increased heat loads can have substantial impacts on populations (Sinervo et al., 2010), they first originate as thermal constraints on individuals that are often mediated by an organism's behavior and physiology (van Beest, Van Moorter, & Milner, 2012; Kendeigh, 1949; Mosauer, 1936). Refuge seeking is a critical behavior that allows many reptile (Attum, Kramer, & El Din, 2013; Lagarde et al., 2012; Mack, Berry, Miller, & Carlson, 2015), mammal (van Beest et al., 2012; Cain, Jansen, Wilson, & Krausman, 2008), and bird (Wolf & Walsberg, 1996; Wolf, Wooden, & Walsberg,^1996^) species to lessen the impacts of extreme heat events or avoid heat stress by exploiting more favorable microclimates available to them. Thermal refuge can be provided by abiotic (i.e., landform, topography; Millar, Westfall, & Delany, 2016) or biotic (i.e., vegetation cover) (Attum et al., 2013; van Beest et al., 2012) landscape features. Importantly, the survival of organisms can hinge on their ability to locate and occupy refuges that modulate extreme heat conditions; accordingly, previous research has identified how fine scale microrefuges can serve as thermal refuge for endotherms and ectotherms (Attum et al., 2013; Carroll, Davis, Elmore, Fuhlendorf, & Thacker, 2015; Lagarde et al., 2012; Wolf et al., 1996). Despite the acknowledged importance of thermal refuge for many species, it remains unclear whether thermal refuges will continue to effectively buffer organisms from future heat extremes (Keppel & Wardell‐Johnson, 2012; Scheffers, Edwards, Diesmos, Williams, & Evans, 2014; Suggitt et al., 2011), or whether suitable buffered conditions will continue to exist (Carroll, Davis, Fuhlendorf, & Elmore, 2016). For example, climate change effects may also induce major shifts in vegetation structure (Breshears et al., 2005, 2009; Kelly & Goulden, 2008) which consequently could alter the availability of thermal cover for species. Therefore, assessing how microhabitats buffer thermal extremes will be important for linking changes in climate to changes in microclimate (Goller, Goller, & French, 2014; Potter, Arthur Woods, & Pincebourde, 2013; Scheffers et al., 2014) and can provide perspectives that are more relevant to organisms than approaches that assess thermal conditions at broader scales (e.g., >1 km) (Gunderson & Leal, 2012; Hannah et al., 2014; Helmuth et al., 2010; Sears et al. 2011). This linkage will be fundamentally necessary for understanding when, where, how, and if organisms will adjust to more extreme temperatures in the future and also for assessing how microhabitats may provide in situ thermal modulation relative to climate change (Keppel et al., 2015; Moritz & Agudo, 2013).

**Figure 1:**
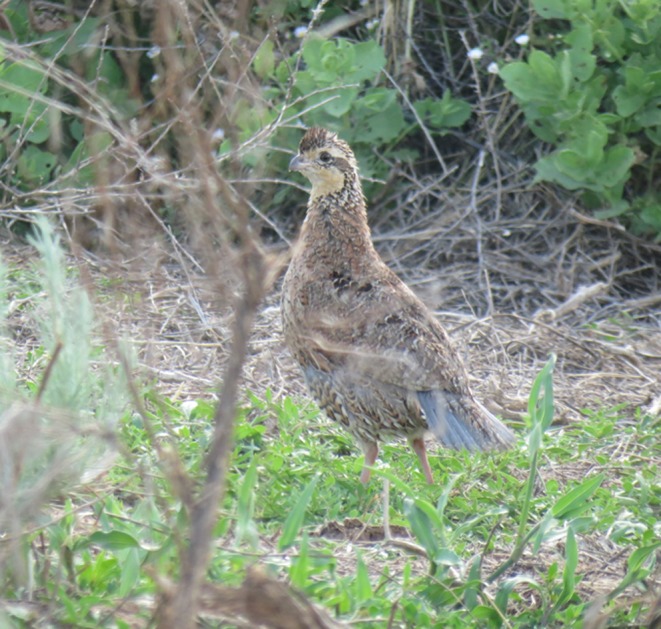
Female northern bobwhite (*Colinus virginianus*) photographed in western Oklahoma, USA

Studies that capture naturally occurring periods of extreme heat in order to assess how climate change may influence endotherms are scarce (Boyles et al., 2011; McKechnie et al., 2012; Parmesan et al., 2000) especially since such events can be logistically or temporally difficult to capture (e.g., episodic heat waves). Nevertheless, the rates of catastrophic die offs and sublethal effects (e.g., fitness costs, constraints on growth and development) are predicted to increase in frequency for many endothermic bird species in the future (Cunningham, Kruger, Nxumalo, & Hockey, 2013; McKechnie & Wolf, 2010). Ground‐dwelling birds may be especially at risk to heat exposure because they inhabit the near‐ground thermal medium, which is subjected to extremely high and variable temperatures (Rosenberg, Blad, & Verma, 1983). Accordingly, models of bird abundance suggest that populations of nonmigratory ground nesting birds are more vulnerable than other avifauna to heat waves and drought (Albright et al., 2010). As a small nonmigratory ground‐dwelling bird species, northern bobwhite (*Colinus virginianus;* hereafter bobwhite) (see Figure 1) are a useful model for assessing microhabitat use during extreme heat events for several reasons (Carroll, Davis, Elmore, & Fuhlendorf, 2015; Carroll, Davis, Elmore, Fuhlendorf, & Thacker, 2015). First, bobwhites are regularly confronted with thermal stress and potentially lethal microclimates due to high heat and solar radiation during summer in the Southern Great Plains of North America (Forrester, Guthery, Kopp, & Cohen, 1998; Guthery, 2000; Guthery, Land, & Hall, 2001). Second, there is only a 4.4°C difference between normal bobwhite body temperature (42.6°C) and lethal temperature (47°C) which requires them to actively dissipate heat both physiologically (i.e., gular flutter) and behaviorally (i.e., occupying refuges and reducing activity) in order to mitigate heat extremes (Guthery, 2000). Third, bobwhite chicks are much more vulnerable to direct solar radiation and high heat than adults, yet annual bobwhite breeding, nesting, and brood rearing cycles temporally overlap with yearly peaks in heat extremes (i.e., summer in North America; Guthery, 2000). Consequently, the susceptibility of bobwhites to high heat exposure across multiple life stages, combined with the occurrence of unusually hot conditions, provides a context from which to examine how organisms may respond to the more extensive and extreme heat associated with predicted climate change.

The climate of the North America's Southern Great Plains is characterized by periodic drought events and heat waves (Arndt, 2003). However, relative to historical records, the high heat that occurred during 2012 in the Southern Great Plains was particularly extreme (i.e., ambient temperature up to 43.4°C). Specifically, 43 days during the summer of 2012 had ambient temperatures (*T*_air_) ≥ 35°C which exceeded the average summer maximum *T*_air_ reported for the region from 2000 to 2014 (33.3°C) (Arnett Oklahoma Mesonet Site; Oklahoma Mesonet, 2016a,b). Therefore, the timing of our study provided a serendipitous opportunity to conduct a natural experiment on the thermal ecology of a ground‐dwelling bird species, specifically bobwhites. It also allowed us to focus on two scales that were directly relevant to two differing life stages (i.e., brood rearing and nonbrood rearing). Our primary objective was to assess potential thermal exposure and bobwhite refuge use compared to thermal conditions on the prevailing landscape during a period of extreme heat. Our secondary objective was to use bobwhites as a model species to estimate thermal buffering at refuge sites and validate future heat load predictions for ground‐dwelling birds. Finally, we aimed to evaluate the capacity of thermal refuges to continue to modulate microclimate in the future.

## 2 MATERIALS AND METHODS

### 2.1 Study area

**Figure 2:**
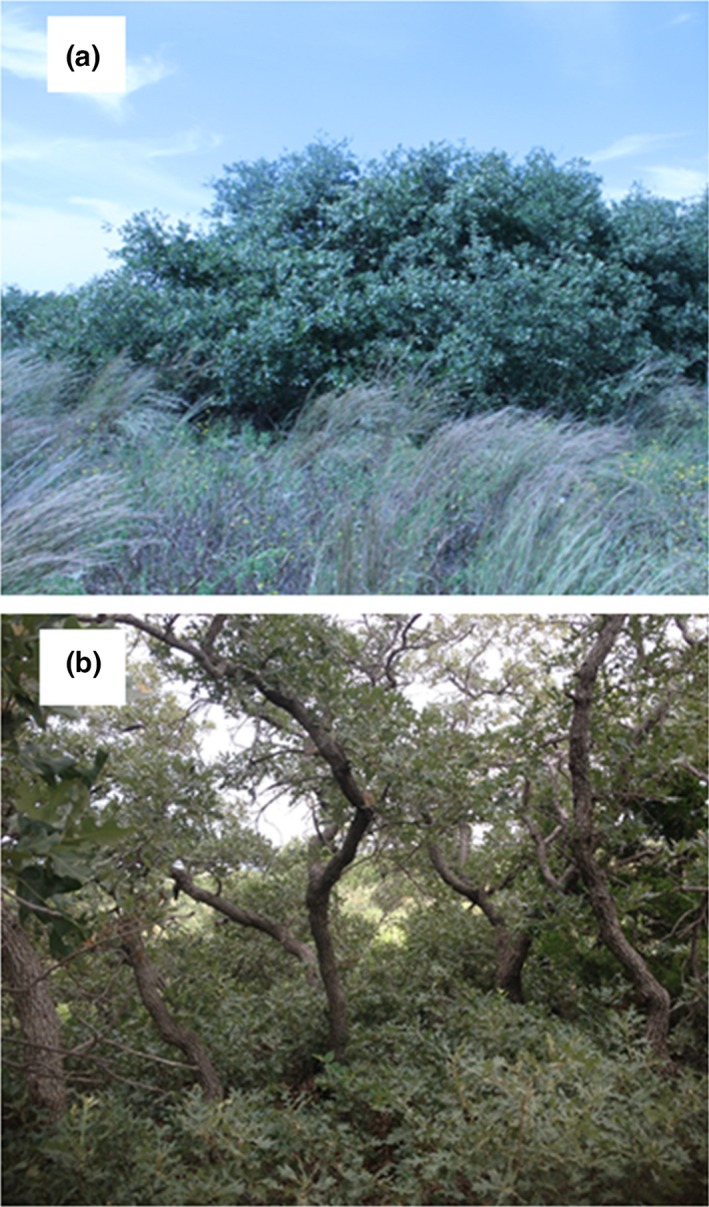
Representation of (a) the exterior and (b) the interior of hybrid shinnery oak patches which provide discrete thermal refuges for northern bobwhites (i.e., adults and broods) during heat extremes at the Packsaddle WMA, Oklahoma, USA, 2012

We conducted our study at Packsaddle Wildlife Management Area (WMA) which is owned by the Oklahoma Department of Wildlife Conservation. The WMA is located in western Oklahoma, USA, and is 7,956 ha in extent. The study area is a mixed‐grass shrub landscape, and sand shinnery oak (*Quercus havardii*) is the most dominant shrub and is a native species that grows to approximately 0.3–1.2 m in height in clonal mottes (i.e., clumped thickets) (Peterson & Boyd, 1998). A hybrid form of sand shinnery oak and post oak (*Quercus stellata*) also occurs patchily in the study area and typically reaches heights well in excess of 1.8 m (Wiedeman & Penfound, 1960), thereby standing much taller than most other plant species on the surrounding landscape (Peterson & Boyd, 1998; Figure 2). Detailed information on the vegetation community of the study area is provided by DeMaso, Peoples, Cox, and Parry (1997).

Located in the Southern Great Plains of the United States, Oklahoma's climate is characterized by highly variable precipitation among years, as well as common drought events and heat waves which often occur in tandem (Arndt, 2003). Although hot and dry conditions can be common in the Southern Great Plains (Arndt, 2003; Rosenberg, 1986), our study year (2012) and period (May–July) captured a comparatively extreme heat and drought event. Specifically, the timing of our study captured intense and frequent bouts of extreme heat. As recorded by on‐site weather stations, the study period underwent 20 days of *T*_air_ ≥39°C which represents the heat stress and hyperthermia threshold for bobwhites (Forrester et al., 1998) and 2 days of *T*_air_ > 43°C which equaled or exceeded the maximum *T*_air_ reported from other field studies on the effects of heat on bird behavior in arid regions of the world (Cunningham, Martin, et al., 2013; Cunningham, Kruger, et al., 2013; Edwards, Mitchell, & Ridley, 2015; Martin, Cunningham, & Hockey, 2015; du Plessis et al., 2012). Moreover, rainfall during the study period (41.7 mm) was 20% of the average from 1994 to 2015 (246.1 mm; Arnett Oklahoma Mesonet Site; Oklahoma Mesonet, 2016a,b).

### 2.2 Capture and radio‐marking

During the winter and spring (February–May) of 2012, we used Stoddard style funnel traps (Stoddard, 1931) to capture adult bobwhites and each captured individual that weighed >130 g was collared with a 6‐g necklace radio‐collar (*n* = 78 individuals; 40 females and 38 males) (Advanced Telemetry Systems, Isanti, MN). We located radio‐marked adults 4–7 times per week by homing (White & Garrott, 2012) to determine bobwhite locations. Our homing technique involved circling telemetered birds at a distance of 10–15 m and recording an estimated distance and bearing. Given that our homing involved circling birds from a distance of 10–15 m, we were able to determine the vegetation patch containing the radio‐marked individual and estimate the birds’ location within each respective patch. The homing technique has been used in a substantial amount of studies on the habitat use of gallinaceous birds (Grisham, Borsdorf, Boal, & Boydston, 2014; Patten, Pruett, & Wolfe, 2011; Winder et al., 2014) and the thermal ecology of gallinaceous birds (Guthery et al., 2005; Hovick, Elmore, Allred, Fuhlendorf, & Dahlgren, 2014), including bobwhite (Carroll, Davis, Elmore, Fuhlendorf, & Thacker, 2015; Guthery et al., 2005). Moreover, it allows for an assessment of refuge use given that bobwhite movement is severely curtailed during hot periods on summer days (Carroll, Davis, Elmore, Fuhlendorf, & Thacker, 2015).

From May to July 2012, we monitored non brood attending adults as well as broods (i.e., chicks associated with a radio‐marked adult) to determine bobwhite habitat use from a combined biotic (i.e., vegetation) and abiotic (i.e., temperature) perspective. We included locations from May in our study period because May 2012 was characterized by extreme drought (i.e., only 0.25 mm of rainfall during the month) and above average temperatures (Arnett Oklahoma Mesonet Site; Oklahoma Mesonet, 2016a,b). To obtain a representation of bobwhite thermal ecology relative to high heat, we obtained radio‐locations from 11:00 to 17:00 hr since these times correspond to peak diurnal heating and therefore potential thermal stress in bobwhites. For each radio‐tracking occasion, we randomly selected radio‐marked birds (i.e., adult or brood with attending adult) for radio‐tracking based on the available pool of marked individuals. Confirmation that adults were accompanied by a brood (≥1 chick) was achieved within 2 days of each radio‐telemetry bout by visually confirming brood presence through flushing, as well as observing chick feces or distraction displays by adults (Carroll, Davis, Elmore, Fuhlendorf, & Thacker, 2015; Taylor & Guthery, 1994).

### 2.3 Thermal sampling

To obtain an index of the thermal characteristics at bobwhite use sites compared to those on the landscape, we measured black bulb temperature (*T*_bb_). *T*_bb_ is a single measurement derived from the effects of multiple environmental variables simultaneously (i.e., ambient temperature, solar radiation and wind; Bakken, Santee, & Erskine, 1985; Campbell & Norman, 1998; Gagge, 1940). Therefore, *T*_bb_ better represents the thermal environment experienced by an organism than ambient temperature (*T*_air_) (Helmuth et al., 2010). We assessed T_bb_ using black bulb thermometers (hereafter, black bulbs) which consisted of steel spheres coated with flat black paint (101.6 mm‐diameter; 20 gauge thickness) that were placed at ground level at each location (Allred et al., 2013; Guthery et al., 2005; Hovick et al., 2014). To measure and record *T*_bb_, each black bulb was fitted with an internally centered *T*_air_ sensor attached to a HOBO U12 data logger (Carroll, Davis, Elmore, Fuhlendorf, & Thacker, 2015; Carroll, Davis, Elmore, & Fuhlendorf, 2015; Hovick et al., 2014).

Although *T*_bb_ recorded by black bulbs does not provide a complete representation of the thermal conditions experienced by bobwhites given that black bulbs do not fully reproduce bobwhite feather composition or color (Dzialowski, 2005), *T*_bb_ measurements do provide an index of heat loads occurring on the landscape and those experienced by bobwhites. We recognize that bobwhites likely experience lower heat loads than those described by *T*_bb_ given that the short‐wave absorptivity of black bulbs (~1) exceeds that of a bobwhite (0.78) (Calder & King, 1974; Guthery et al., 2005). Nevertheless, measuring *T*_bb_ provides a standardized way to index thermal environments and has been a commonly used methodology in the thermal ecology of galliforms (Carroll, Davis, Elmore, Fuhlendorf, & Thacker, 2015; Carroll, Davis, Elmore, & Fuhlendorf, 2015; Guthery et al., 2005; Hovick et al., 2014). Therefore, our objective was to obtain an index of thermal environments exploited by bobwhites compared to those available on the prevailing landscape.

Black bulbs were deployed at adult (*n* = 40) and brood locations (*n* = 37) observed from 11:00–17:00 hr on the day following radio‐tracking assuming that weather conditions were similar to those on the day that location was observed (Carroll, Davis, Elmore, Fuhlendorf, & Thacker, 2015; Guthery et al., 2005). Given that our study was conducted during a summer characterized by historic high heat, weather conditions from day to day were relatively uniform. We used 11:00–17:00 hr to categorize refuge use as bobwhite adult and brood movement has been shown to be substantially reduced or ceased during the heat of the day as they loaf and seek thermal refuge (Carroll, Davis, Elmore, Fuhlendorf, & Thacker, 2015). To assess the thermal landscape, we also conducted thermal sampling at 104 stratified random points distributed across the study area. We obtained stratified random points (i.e., based on proportion of vegetation types) using a vegetation layer in ArcGIS 10.3 (Environmental Systems Research Institute, Redlands, California, USA) that was created using 125 training points and an additional 215 used for ground truthing. A black bulb was placed at each radio‐marked non‐brood attending adult and brood attending adult location and *T*_bb_ was recorded at 15‐min intervals for 24 hr (*n* = 5,068). We then averaged *T*_bb_ for each hour from 11:00 to 17:00 hr (*n* = 1,267) so that it would match hourly ambient temperature and solar radiation that was simultaneously recorded at a weather station (2 m above ground) located in the study area. We conducted thermal sampling during similar average hourly *T*_air_ conditions at adult (range: 18.33–41.85°C), brood (range: 20.97–43.44°C), and random sites on the landscape sites (range: 23.92–43.44°C; Table 1).

**Table 1:** Range of ambient temperature (*T*_air_) and black bulb temperature (*T*_bb_) sampled at northern bobwhite adult refuge sites (*n* = 40), brood refuge sites (*n* = 37), and stratified random sites (*n* = 104) (11:00–17:00 hr) at the Packsaddle WMA, Oklahoma, USA, 2012

Site	*T*_air_ Range (°C)	*T*_bb_ Range (°C)	*T*_bb_ Mean (±SE)^a^
Adult	18.33–41.85	23.12–61.63	39.33 (±0.46)^A^
Brood	20.97–43.44	24.92–58.71	42.18 (±0.40)^A^
Random	23.92–43.44	30.17–72.43	52.23 (±0.27)^B^

a Different letters denote significant differences (*p* < .05) (Tukey's multiple comparisons).

Before analysis, we classified days with maximum *T*_air_ < 35°C as “moderate,” *T*_air_ ≥ 35 – <39°C as “hot,” and *T*_air_ ≥ 39°C as “extreme.” We chose these categories because they represent biologically relevant thresholds for bobwhites. Specifically, 30–35°C is considered to be thermoneutral for bobwhites (Lustick, 1972), ≥35 – <39°C is considered thermally stressful but not hyperthermic (Guthery, 2000), and ≥ 39°C represent hyperthermic conditions (Guthery, 2000; Guthery et al., 2001). Specifically, at operative temperatures of 39°C, the rate of heat removal is exceeded by heat gain in bobwhites (Guthery, 2000) and this physiological threshold has been used for the analysis of thermal data and climate projections for galliforms such as greater prairie chickens (Hovick, Elmore, Fuhlendorf, & Dahlgren, 2015) and bobwhites (Carroll, Davis, Elmore, & Fuhlendorf, 2015; Guthery et al., 2005).

### 2.4 Vegetation sampling

To understand how bobwhites utilize vegetation patches on the landscape during potentially thermally stressful periods (11:00–17:00 hr), we sampled vegetation characteristics at adult and brood locations. For comparisons with vegetation at bobwhite locations, we also conducted vegetation sampling at points derived from our stratified random sampling approach using ArcGIS 10.3 (Environmental Systems Research Institute, Redlands, California, USA). Therefore, the stratified random sampling points allocated for vegetation sampling were representative of available vegetation in the study area.

To assess canopy structure and coverage, we measured the angle of obstruction (°) in eight compass directions (cardinal and sub‐cardinal) at each location (Kopp, Guthery, Forrester, & Cohen, 1998). To accomplish this, a 2‐m pole with a digital carpenter's level attached to it was aligned with the top of nearest vegetation in each of the eight directions (Kopp et al., 1998). We also centered a 0.5 m^2^ quadrat (modified from Daubenmire, 1959) over the estimated bird location or random location to estimate percent bare ground, litter, grass, forb, and woody cover. Vegetation height at each sampling point was classified into categories of <1 m, ≥1‐ <2 m, and ≥2 m given that bobwhites have been shown to utilize varying vegetation heights throughout the day at adult (Hiller & Guthery, 2005) and brood locations (Carroll, Davis, Elmore, Fuhlendorf, & Thacker, 2015). To better inform the potential management of thermal space and because thermal refuge selection by birds can be species specific (Martin et al., 2015), we also we recorded the dominant species at each location (e.g., hybrid shinnery oak, sand plum, etc.).

### 2.5 Analyses

To assess bobwhite site selection relative to the thermal landscape, we analyzed T_bb_ as a dependent variable among all bobwhite locations (i.e., adult and brood) and stratified random points as an independent variable using a one‐way analysis of variance (ANOVA) (Zar, 1984). Additionally, we compared hourly mean differences in *T*_bb_ for moderate (maximum *T*_air_ < 35), hot (maximum *T*_air_ ≥ 35 – <39°C), and extreme (maximum *T*_air_ ≥ 39°C) days using ANOVA.

Potential differences in thermal buffering of *T*_bb_ at bobwhite locations and stratified random sites were evaluated by calculating the difference between mean hourly *T*_bb_ measurements at each location and mean hourly *T*_air_ recorded at onsite weather stations (*T*_bb_−*T*_air_; Carroll, Davis, Elmore, Fuhlendorf, & Thacker, 2015; Carroll, Davis, Elmore, & Fuhlendorf, 2015). The resulting values were tested for differences between refuge sites and random sites using ANOVA (Zar, 1984). We also compared angle of obstruction and percent cover among bobwhite use sites (i.e., adult and brood locations) and stratified random sites for each daily *T*_bb_ category (i.e., moderate, hot, and extreme) using ANOVA. Differences were deemed significant at the *p* < .05 level.

## 3 RESULTS

### 3.1 Thermal environments

**Figure 3:**
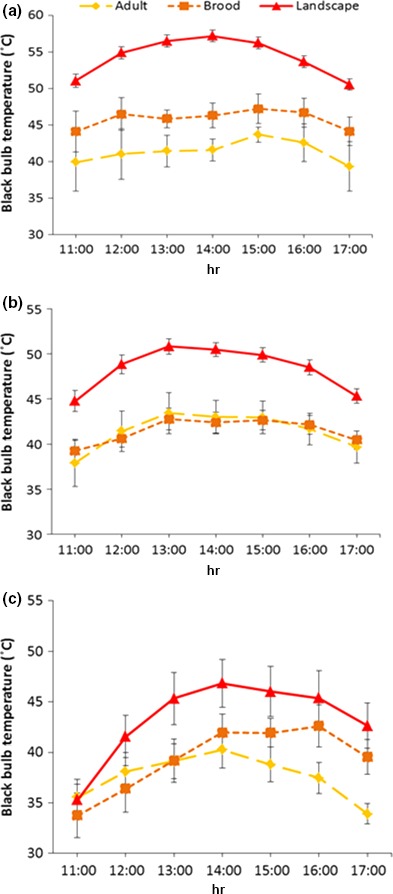
Black bulb temperature (*T*_bb_) (±SE) averaged by hour among adult refuge sites, brood refuge sites, and stratified random sites on (a) extreme (≥39°C), (b) hot (≥35 – <39°C), and (c) moderate (<35°C) days at the Packsaddle WMA, Oklahoma, USA, 2012

**Figure 4:**
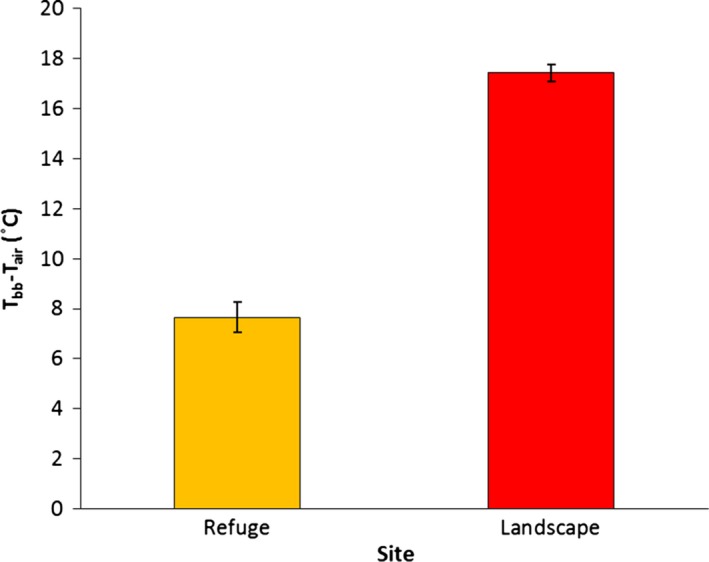
Mean differences between hourly black bulb temperature (*T*_bb_) and ambient temperature (*T*_air_) measurements (*T*_bb_−*T*_air_) (±SE) among refuge sites (i.e., adult and brood) and random sites on extreme (*T*_air_ ≥ 39°C) days (*n* = 16) at the Packsaddle WMA, Oklahoma, USA, 2012

We found that *T*_bb_ at stratified random points on the landscape could potentially reach extreme temperatures (e.g., 72°C), and that mean *T*_bb_ exceeded 50°C from 11:00 to 17:00 hr (Table 1). Despite these heat extremes occurring throughout the study area, mean *T*_bb_ at adult and brood locations remained at least 10°C cooler on average than random sites on the landscape (Table 1). These differences were observed on moderate (*F*_2,325_ = 52.49, *p* < .001) as well as hot days (*F*_2,340_ = 87.78, *p* < .001), but were most pronounced on extreme days (*F*_2,592_ = 101.50, *p* < .001) when thermal buffering for bobwhites was likely most critical (Figure 3). Additionally, *T*_bb_ at random sites on the landscape averaged 57.17°C at 14:00 hr and was greater than 50°C for the entire refuge period (11:00–17:00 hr) on extreme heat days (Figure 3). Despite the landscape being inundated with extreme *T*_bb_, adult and brood refuge sites provided thermal environments that were on average 13.98°C and 8.45°C cooler than random sites on landscape, respectively (maximum mean differences of up to 16.51°C and 10.88°C, respectively; Figure 3). We also found that differences between *T*_bb_ and *T*_air_ (*T*_bb_−*T*_air_) at refuge sites (adult and brood sites) were substantially less than at random sites (*F*_2,585_ = 96.31, *p* < .001; Figure 4) and bobwhite refuge sites moderated thermal conditions by more than twofold compared to the landscape on extreme days (*F*_2,1263_ = 265.7, *p* < .001; Figure 4). Although refuge locations provided microclimates that were much less extreme than those on the landscape, mean *T*_bb_ at refuge locations regularly exceeded 39°C (i.e., hyperthermic level in bobwhites), with temperatures peaking at 15:00 at adult (43.48°C) and brood sites (47.22°C) on extreme days (Figure 3).

### 3.2 Vegetation characteristics

Vegetation heterogeneity (i.e., patchiness of structure and canopy coverage) influenced the thermal patterns that we observed; specifically, the use of different thermal environments at refuge sites compared to random sites. For example, bobwhite refuge sites were characterized by greater angle of obstruction than at random sites (*n* = 181; *F*_2,175_ = 84.79, *p* < .001) (Figure 2). Specifically, mean (±SE) angle of obstruction (i.e., angle of vertical and overhead vegetation cover) was more than twofold greater at adult (73.45 ± 1.75) and brood refuge sites (79.38 ± 1.13) than at random sites (35.21 ± 2.44). Additionally, adult and brood refuge sites afforded two‐ and threefold greater percent woody cover than at random sites, respectively (*F*_1,175_ = 46.43, *p* < .001). We also observed greater percent litter at bobwhite locations (i.e., adult and brood) than random sites (*F*_2,175_ = 17.98, *p* < .001) as well as greater grass cover at brood locations than random sites (*F*_2,175_ = 8.79, *p* < .001) and less bare ground cover at adult locations than at random sites (*F*_2,175_ = 4.33, *p* < .05). No significant differences were observed for angle of obstruction or percent bare ground, litter, grass, forb, or woody cover between adult and brood refuge sites (*p* > .05). Moreover, angle of obstruction differed across temperature categories (*F*_2,71_ = 12.47, *p* < .001) and was greater at bobwhite locations on days with *T*_air_ of ≥ 35‐ <39°C and ≥39°C than days with *T*_air_ < 35°C. However, we found no differences in percent bare ground, litter, grass, forb, or woody cover at bobwhite locations among daily *T*_air_ categories.

We observed that the percentage of bobwhite locations in vegetation cover ≥2 m tall was 56.25%, 82.75%, and 100% on moderate, hot, and extreme days, respectively; despite that only ~7% of the landscape consisted of this cover type (Table 2). Conversely, herbaceous cover comprised approximately 50% of the study area yet none of the bobwhite locations occurred in this cover type from 11:00 to 17:00 hr on moderate, hot, or extreme days (Table 2). Tall hybrid shinnery oak mottes accounted for 63% of bobwhite refuge sites on extreme days.

**Table 2:** Vegetation types utilized by northern bobwhites at refuge sites (11:00–17:00 hr) on moderate (<35°C) (*n* = 32), hot (≥35 – <39°C) (*n* = 29) and extreme (≥39°C) (*n* = 16) days compared to landscape vegetation availability at the Packsaddle WMA, Oklahoma, USA, 2012

Locations	Cover Type (%)		
	Tall woody	Low woody	Herbaceous
<35°C	43.75	56.25	0.00
≥35 – <39°C	82.75	17.25	0.00
≥39°C	100	0.00	0.00
Landscape Availability	6.78	33.97	50.06

## 4 DISCUSSION

By capturing periods of high ambient temperatures which represented extreme heat for our study region and thermal stress for our study species, we demonstrate how a local resident species responds to heat extremes which approximate conditions that are predicted to become more common in the future (United States Global Research Change Program 2014). Our results agree with findings from previous studies that have shown the thermal importance of tall woody cover for ground‐dwelling birds (Goldstein, 1984; Martin et al., 2015; McKechnie et al., 2012), but most importantly, showcases how tall woody cover functions as critical islands of thermal refuge during extreme heat. Tall woody cover provided refuge from *T*_bb_ that exceeded lethal thresholds for bobwhites (Guthery, 2000; Guthery et al., 2001) as well as most biota (i.e., *T*_bb_ > 50°C) (Calder & King, 1974). Additionally, the cooler microclimates observed at refuge sites compared to the surrounding landscape (i.e., 16.51 and 10.88°C cooler at adult and brood sites, respectively) are biologically significant given that even small differences in temperature (i.e., 2–4°C) on the landscape become increasingly impactful as the gap between body temperature and environmental temperature is lessened in birds (Ricklefs & Hainsworth, 1968, 1969). Nevertheless, the high *T*_bb_ observed within refuge sites (≥39°C) provides further evidence that ground‐dwelling, nonmigratory species such as bobwhite may be highly vulnerable to climate change effects as extreme temperatures become more common (Carroll, Davis, Elmore, Fuhlendorf, & Thacker, 2015; McKechnie & Wolf, 2010).

Behavioral plasticity can be an important mechanism for allowing organisms to adjust to changing conditions, especially climatic extremes (Allred et al., 2013; Wolf et al., 1996). However, the effectiveness of such adjustments is contingent upon locating more favorable conditions on the landscape. In contrast to some reptile species that have the option of avoiding extreme heat by seeking underground burrows (Beck & Jennings, 2003; Mack et al., 2015), bobwhites are limited to seeking above‐ground refuges which are typically comprised of woody vegetation (Carroll, Davis, Elmore, Fuhlendorf, & Thacker, 2015; Forrester et al., 1998). Despite the lack of differences in vegetation structure that we observed between adult and brood sites, we found that mean *T*_bb_ at adult sites remained cooler than brood sites by up to 5.4°C on the most extreme days. One possibility for this difference is that adults without broods may have exploited thermal environments at different scales than adults with broods, even within similarly structured vegetation patches. For example, despite their greater vulnerability to solar radiation (Guthery, 2000), chicks can obtain suitable microclimates at much finer scales than adults (e.g., under a single leaf). Moreover, adults without broods are unconstrained from additional predator avoidance associated with brood attendance which may have allowed them to select the most favorable microclimate in their immediate proximity (i.e., within a given refuge patch). These findings demonstrate how spatial and temporal scales associated with specific life stages can influence thermal exposure (Angilletta, 2009; Potter et al., 2013), a topic that should be considered more thoroughly in the future species conservation efforts.

Although shade buffers ground‐level heat loads (Rosenberg et al., 1983), we observed that birds in refuges (i.e., brood attending and non‐brood attending adults) were still regularly subjected to average T_bb_ exceeding 39°C (i.e., potentially hyperthermic conditions; Guthery, 2000) for at least 5 hr daily on extreme days. These findings further fortify predicted concerns that eventually the capacity of tall woody cover to modulate extremes (primarily by blocking solar radiation) may be offset by rising ambient conditions predicted with climate change (Carroll et al., 2016; McKechnie et al., 2012). In such a scenario, current thermal refuges of ground‐dwelling birds could become increasingly pervaded with unsuitable or lethal conditions. The possibility for reduced functionality of tall woody cover as refuge sites has direct implications for bobwhite populations in the hottest and driest part of their distribution whose persistence is suggested to be intrinsically linked to the presence of tall woody cover (Guthery, 2000). In such cases, individuals would likely undergo increased evaporative water loss and heat stress on a more regular basis, whereas populations may be subject to inhibited reproduction more frequently given that each have been associated with heat extremes (Guthery et al., 2001).

By 2100, summers are predicted to become hotter with more frequent and extreme heat waves and drought in the Southern Great Plains (United States Global Research Change Program (USGCRP), National Climate Assessment, 2014). Currently, an average number of 7 days per year exceed 37.8°C in the Southern Great Plains but this number is expected to increase by fourfold, and thus, organisms in the region will be faced with higher temperatures more frequently (United States Global Research Change Program (USGCRP), National Climate Assessment, 2014). For example, recent studies have shown that time spent during thermally stressful conditions is predicted to increase two‐ to fourfold for greater prairie‐chickens (*Tympanachus cupido*) in the southern Flint Hills, USA (Hovick et al., 2014). Moreover, substantial increases in ground‐level heat load exposure are predicted for bobwhites in the Southern Great Plains of North America (Carroll, Davis, Elmore, Fuhlendorf, & Thacker, 2015) and increased exposure to high heat is also predicted to influence the fitness of birds on other continents, including Australia (McKechnie & Wolf, 2010) and Africa (Cunningham, Martin, & Hockey, 2015; Cunningham, Martin, et al., 2013; du Plessis et al., 2012). Interestingly, we found that the *T*_bb_ observed in our study approached and in some cases exceeded predicted *T*_bb_ under low emission climate scenarios (i.e., 15:00 and 17:00 hr at brood sites; Carroll, Davis, Elmore, Fuhlendorf, & Thacker, 2015). Although these findings provide a lens from which to approximate thermal conditions that may be associated with climate change, the extreme heat captured in our study generally indicated lower *T*_bb_ than is predicted at ground level in the future, especially under high emission scenarios (Carroll, Davis, Elmore, Fuhlendorf, & Thacker, 2015). Therefore, ground‐dwelling birds may face future microclimates that are substantially more extreme than those experienced during current heat waves and this increased potential for thermal risk should be acknowledged in the future planning and conservation practices.

Our study demonstrates that while capturing periods of thermal extremes tends to be logistically difficult in ecological research (Boyles et al., 2011), doing so can provide insight into how organisms may respond to extremes and serve as a proxy for forecasting future behavior and thermal exposure. By assessing temporal and spatial scales similar to those experienced by a small ground‐dwelling bird during two life stages, we were able to depict conditions that may be faced under future extremes that would not be possible with broader scale climate modeling. Future research should focus on evaluating how changes in microclimates within thermal refuges may influence species responses and also attempt to identify thresholds at which thermal refuges may no longer provide tolerable or survivable conditions (Scheffers et al., 2014). Such information will be critical for not only understanding organism responses to extremes but also quantifying the thermal refugia capacity of landscapes and predicting future population persistence.

## CONFLICT OF INTEREST

None declared.
